# The influence of distributive justice on job attitudes and life satisfaction of hotel workers

**DOI:** 10.1016/j.heliyon.2024.e25961

**Published:** 2024-02-09

**Authors:** Christopher Mensah, Edem M. Azila-Gbettor, Melody E. Appietu

**Affiliations:** a Department of Hospitality and Tourism Management, Ho Technical University, Ghana; b Department of Management Sciences, Ho Technical University, Ghana

**Keywords:** Distributive justice, Job involvement, Job satisfaction, Life satisfaction, Hotel employees, Accra

## Abstract

This study uses the social exchange and spillover theories to examine the interrelationships between distributive justice, work attitudes, and life satisfaction of hotel employees in Accra, Ghana. Paper-and-pencil questionnaires were used to collect data from 321 respondents after which descriptive statistics and partial least square structural equation modelling were used to analyse the data. Distributive justice and work attitudes were positively related to the life satisfaction levels of hotel employees. This study adds to the scant literature on how workplace goings-on spillover to affect the life satisfaction of hotel workers. Theoretical and practical implications of the results are discussed.

## 1 Introduction

Management of human resources within the hospitality industry continues to attract the keen interest of practitioners and researchers [[1], [2], [3]] because of the industry's significant contributions to economic growth in several countries [4]. In the service industry, scholars have posited that people contribute immeasurably to firms competitive advantage [[5], [6], [7]] and success [8]. Efficient operations of hotels depend largely on effective and strategic management of human resources [9]. According to Kandampully et al. [10], human resources are especially important in labour-intensive service industries like the hotel sector, where employees and guests interact a lot at the service interface. In sum, appropriate management of human resources keeps employees happy, ensures employees behave appropriately, and helps maintain the excellent reputation of the business.

Organizational justice, defined as an employee's perception of fairness [11], is considered an essential ingredient for effective human resource management because it drives employees' positive opinions about their jobs and perceived workplace meaningfulness [12]. In addition, distributive justice provides an interpretive lens for how people interact at their workplaces [13,14]. Distributive justice, the focus of this study, addresses the issue of whether the outcome received by workers is perceived as fair instead of the absolute level of the outcome [13]. Distributive justice is hinged on moral infrastructure and encompasses principles that regulate societal resources [15]. Organ [16] posits that an employee's decision to display appropriate behaviour may be a function of how he or she perceives being treated fairly. Similarly, research evidence from Fox et al. [17] and Lilly [18] has demonstrated that employees respond to workplace unfairness with a variety of negative behavioural responses, including resistance, theft, and withdrawal..

Available evidence suggests employees’ perceptions of distributive justice have been a significant predictor of optimistic work attitudes and behaviours, including job involvement, job satisfaction, life satisfaction, and organizational commitment [**2, 19–23**], yet there is a paucity of literature from the perspective of hospitality industries in developing countries. Specifically, studies on the link between distributive justice and the four work-attitude variables of job involvement, job satisfaction, life satisfaction, and organizational commitment in Ghanaian hotels are limited [19,20]. Besides, the contribution of distributive justice to job outcomes and work attitudes has been debated, and it is uncertain whether its impact remains stable across employees [21].

In the Ghanaian hospitality context, which is largely dominated by lower-rated hotels, anecdotal evidence suggests employee welfare or fairness is a major issue. Similarly, there is a tacit consensus among practitioners and policymakers that employee job involvement, life satisfaction and commitment within the Ghanaian hotel sector have been overlooked. This is partly because most hotels use an informal management style and neglect efficient human resource management procedures [22]. Despite the significance of work attitudes to several organizational outcomes, the interrelationships among job involvement, life satisfaction, employment commitment and organizational justice among Ghanaian hotel workers are less understood. The study of the above listed work attitudes is considered important in the hospitality industry since Min et al. [23] and Liu et al. [24] posited that committed and involved employees are organizational assets, which ultimately increase motivation and enhance productivity. Similarly, employees’ job and life satisfaction has been identified as a significant indicator of desirable organizational outcomes, including quality service delivery, extra-role service behaviours and cooperation at team and individual levels [25,26]. Meanwhile, given that life satisfaction is an important social aspiration, investigating distributive justice as a driver of life satisfaction is a worthwhile research endeavour, given that an earlier study solely focused on correctional staff [27]. It is therefore imperative to evaluate how justice perception relates to employees' perceptions of job involvement, job satisfaction, and organizational commitment of hotel workers to provide a basis for comparison across different work populations. Consequently, the purpose of this study is to assess the path relationships relative to distributive justice, job involvement, job satisfaction, life satisfaction and organizational commitment. The specific research objectives of the study are to:

1. examine the effect of distributive justice on job involvement, job satisfaction, life satisfaction and organizational commitment.
2. explore the relationship between job involvement, job satisfaction and life satisfaction.
3. assess the relationship between job satisfaction and life satisfaction.
4. examine the association between organizational commitment and job involvement, job satisfaction and life satisfaction.

The study makes the following contributions to the hospitality literature. Firstly, the study contributes to the organizational justice literature by examining distributive justice from the perspective of Ghana, a concept that has received less attention. The study further offers evidence to support a positive relationship between distributive justice and job involvement, job satisfaction, life satisfaction and organizational commitment within the context of hospitality firms in a developing country. This finding has added a different cultural perspective to the available literature on employee fairness perceptions regarding the distribution of resources and job outcomes. The study reveals managers can support employees in engaging in positive work behaviours when they exhibit behaviours considered fair by employees. Finally, the study offers managers meaningful experiential recommendations on how to improve the delivery of distributive justice in their organizations.

## 2 Literature review

### 2.1 Distributive justice

Distributive justice is based on the exchange principle or equity theory [28,29] and is assumed to be related to individuals' ‘‘ends’’ [30]. Distributive justice refers to perceived fairness in the distribution and allocation of organizational outcomes [13,31]. It entails an employee's awareness after comparing his/her outcomes with those of others [32]. Perceptions of unfair outcomes trigger employee anger, frustration and resentment, which in turn negatively affect job attitudes [33]. Employees who perceive unfairness are likely to report higher levels of psychological pressure and engage in aggressive workplace behaviours [34,35]. On the other hand, justly treated workers are likely to demonstrate meticulousness at work, adhere to workplace policies and act unselfishly towards others and their organizations [36]. Lin et al. [37] also opined that justly treated employees trust their organization to protect them. In the context of the hospitality industry, research has demonstrated that employee justice perceptions influence several job outcomes [19,38].

### 2.2 Job involvement

Lodahl and Kejner [39] offered the earliest definition of job involvement as “the level to which an employee is identified psychologically with his/her job or the importance of a job in his/her total self-image.” Paullay et al. [40] defined job involvement as a psychological condition where an employee is “cognitively preoccupied with, engaged in, and concerned with one's present job.” Job involvement signifies an attitude towards work [41] that influences the quality of both organizational and individual outcomes [42]. Generally, employees with a high level of job involvement feel competent and become engrossed in their work [43]. In addition, a high level of job involvement reduces turnover and absenteeism but enhances employee productivity [44] and subsequently drives employees to invest considerable effort towards the achievement of organizational objectives [45,46]. On the other hand, lowly involved employees experience burnout, chronic stress, low motivation and low productivity [47]. In the view of Lambert et al. [18], job involvement is an important construct that requires further investigation, and this position is supported by an earlier opinion by Brown [48], suggesting that a deep understanding of the determinants of job involvement will enrich the workplace experiences of employees.

### 2.3 Job satisfaction

Different views exist on the definition of job satisfaction. These perspectives account for the varied definitions of job satisfaction in the extant literature. Job satisfaction is usually defined as either positive or negative feelings about many facets of work, including the nature of the work, supervision style, relationships with coworkers, and job security, among others [49,50]. According to Wright [51], job satisfaction is a consequence of balancing the expectations, needs, or actual outcomes of the position with the benefits of the job. Locke [52] defined job satisfaction as the pleasurable emotional state resulting from the appraisal of one's job as achieving or facilitating the achievement of one's job values. According to Bernstein and Nash [53], job satisfaction has both behavioural effects such as excitement, anxiety, feelings of happiness, boredom, and emotional effects including late working, tardiness, early arrival, and faking illness components. Job satisfaction affects job performance [54] and organizational citizenship behaviour [2], among many others.

### 2.4 Life satisfaction

Life satisfaction is an individual's complete cognitive assessment of life [55] and an established indicator of well-being [56] or quality of life [57]. Life satisfaction is defined as happiness, overall satisfaction, and subjective well-being of which an individual is aware [58]. Erdogan et al. [59] described life satisfaction as a cognitive assessment of the global degree of satisfaction an individual has with her/his overall life. Life satisfaction is related to reciprocal better treatment of colleagues and oneself [59]. Similarly, individuals with high life satisfaction levels have been associated with better health conditions [60] and a lower risk of mortality [61,62]. Life satisfaction is an important psychological construct that affects employees' emotions, with consequences for employee creativity [63] and job performance [64]. As observed by Erdogan et al. [59], the association between life satisfaction and organizational outcomes is an understudied research question deserving additional empirical attention. Almost eight years after the observed research gap, the effects of work attitudes on the life satisfaction of hotel employees remain unexamined.

### 2.5 Organisational commitment

Organizational commitment relates to an employee's state of mind [65], denoting the degree of support and loyalty that employees demonstrate towards their organization [66]. Dee et al. [67] defined organizational commitment as a person's intention to devote themselves to and be loyal to the organization. Dogan and Kilic [68] suggest that extremely committed personnel show considerable trust in the values and goals of their organizations. Additionally, committed employees are helpful to their coworkers [69] and display fitting behaviours that facilitate organizational development [70], as well as lowering employee intentions to quit [71,72].

### 2.6 Theoretical framework

Two theories, including the social exchange theory (SET [73]) and the spillover theory (ST [74]) were used to explain the expected relationship between distributive justice and employee outcomes among hotel employees.

SET theory is conceptualised as a reciprocal system of interaction that operates through the exchange of rewards to facilitate value exchange [73]. The concept of SET is founded on the idea that the establishment of a relationship between two individuals is facilitated by a systematic evaluation of the costs and benefits involved [[75], [76], [77]]. The SET framework emphasizes the principle of “reciprocity” as a fundamental guideline for social behaviour [78,79]. It suggests that social relationships are deliberate actions driven by the advantages derived from the interactions one has with others [80]. Thus, under the “reciprocity rule,” a caring behaviour leads to a return of favour, to attain a psychological balance between two individuals [81]**.**

Spillover theory explains how transmission across two domains positively (positive spillover) and negatively (negative spillover) affects each other [82]. Spillover theory insists that a person's attitudes, emotions, skills, and behaviours in one domain flow into the other and vice versa, and it can occur in both positive and negative ways [74,83]. Positive spillover occurs when positive experiences or attributes in one domain enhance those in the other, leading to increased well-being and performance. On the other hand, negative spillover refers to the transfer of negative experiences or attributes between domains, which can lead to decreased well-being and performance.

The two theories focus on the examination of human behaviour, attitudes, and decision-making within social and organisational settings. SET underpins hypotheses H1–H4, which explain how perceptions of distributive justice influence hotel employees job attitudes and life satisfaction, and hypotheses H8–H10, which explain how hotel employees' evaluation of their organizational commitment influences their overall job attitudes and satisfaction and life satisfaction. ST supports hypotheses H5–H7 and elucidates how positive experiences and attitudes about employee job involvement can spill over to influence job satisfaction and, in turn, life satisfaction.

Within the framework of this study, these theories interact by influencing the perceptions and attitudes of hotel workers. SET serves as a fundamental framework for comprehending the impact of distributive justice on diverse job attitudes and organisational commitment which have an influence on the holistic state of well-being. The spillover theories illustrate how job-related attitudes have the potential to extend beyond the workplace and impact an individual's overall life happiness. Overall, the integration of these theories allows for a comprehensive understanding of the complex relationship between job attitudes, life satisfaction, and overall well-being in the context of hotel workers.

### 2.7 Distributive justice, job involvement, job satisfaction, life satisfaction and organisational commitment

Emerging literature has confirmed distributive justice as a positive predictor of many workplace outcome variables, including job involvement, job satisfaction, life satisfaction and organisational commitment. For example, a study involving 110 workers at a teaching hospital in Lomé confirmed a positive and significant relationship between distributive justice and job involvement [84]. Ahmadi [85] reported a similar finding in a study involving 140 respondents from the Iranian Customs Affairs Organization.

With regard to job satisfaction, Appaw-Agbola et al. [19] studied 400 employees of family-owned hotels in Accra, Ghana, and found distributive justice to account for significant variance in employees' job satisfaction. Similarly, results from another Ghanaian study conducted by Abekah-Nkrumah and Atinga [86] among 247 health workers revealed that distributive justice was a predictor of job satisfaction. However, in Fulford's [38] study of employees of a major casino hotel located in Las Vegas, distributive justice was unrelated to job satisfaction, like in the study of Khan et al. [87].

Results of a study examining the association between distributive justice and life satisfaction among 272 correctional staff in a Midwestern state in the USA found that employees’ perceptions of distributive justice positively affected their life satisfaction [88]. Lucas et al. [89] also found a similar outcome in their study of predominantly 197 Caucasian urban residents of Michigan, in the USA. Within the hospitality literature, the influence of distributive justice on life satisfaction has attracted limited empirical investigation.

Finally, Ogechukwu et al. [90] conducted a study involving 550 staff from eight ministries in the Rivers State civil service in Nigeria and found a positive and significant relationship between distributive justice and organizational commitment. Chang [91] examined 411 Korean employees working for 14 corporations and found that distributive justice related positively to organizational commitment. Similarly, Lambert et al.‘s [92] study of 322 correctional staff in the USA reported a significant positive relationship between distributive justice and organizational commitment.

The relationship between distributive justice and employees' job involvement, job satisfaction, and organizational commitment can be explained using the SET [73,93]. The tenet of SET emphasizes “exchanges and reciprocity” as a rule of social behaviour. According to the “reciprocity rule,” caring will result in a favour being returned in order to maintain psychological equilibrium between two parties **[86**]. The focus of distributive justice is a fair allocation of the costs and rewards of social cooperation among many people with conflicting demands and claims. In this current study, when employees perceive that the hotels are fair in the distribution of organization resources and outcomes, they positively evaluate and become involved with their jobs, as well as showing affective behaviour towards the hotel. In this regard, a positive relationship is expected between distributive justice and job satisfaction, job involvement, and the organizational commitment of hotel employees. Similarly, the association between distributive justice and life satisfaction can be explained using the spillover theory. According to the spillover theory, a person's attitudes, feelings, skills, and behaviours in one domain flow into their conduct in the other, and vice versa [74]. In this case, the affective and behavioural experiences of workers are likely to spill over into non-work life situations. On this premise, employee perceptions of fairness in the distribution of organization resources are likely to spill over to affect non-work domain conditions such as life satisfaction perception. When employees perceive that their employers are treating them fairly with the sharing of resources, they are likely to report higher levels of life satisfaction. Following the above empirical studies, it was hypothesized that:

H1 There is a positive correlation between distributive justice and job involvement.

H2 There is a positive correlation between distributive justice and job satisfaction.

H3 There is a positive correlation between distributive justice and life satisfaction

H4 There is a positive correlation between distributive justice and organizational commitment.

### 2.8 Job involvement, job satisfaction and life satisfaction

Evidence exists in the extant literature pointing to a positive and significant association between job involvement and job satisfaction [[94], [95], [96]]. For example, highly involved employees are more likely to be satisfied with their jobs compared to less involved employees [97]. Furthermore, several studies found job satisfaction to be an important variable in increasing workers’ sense of self-pride, focus on work, accomplishment and independence, leading to improvements in job involvement [98,99]. Finally, Varshney [96] examined 256 employees selected from six manufacturing companies in India and found a positive association between job involvement and job satisfaction.

Empirical evidence on the relationship between job involvement and life satisfaction is scarce in the organizational literature, except for those conducted on prison staff. Lambert et al. [100] studied the staff of two Chinese prisons and reported a positive and significant association between job involvement and life satisfaction. Similarly, another study involving 120 correctional staff surveyed in 2016 using a cross-sectional design found that greater levels of job involvement were associated with greater satisfaction with life [100]**.** We hypothesized that, on the basis of the affective work-life spillover theory [101], employee job involvement would positively correlate with job and life happiness. This phenomenon is conceivable since their level of job involvement is likely to affect an employee's mood and attitude, which will translate into a satisfactory level of job and life. Consequently, the following hypotheses were developed:

H5 There is a positive correlation between job involvement and job satisfaction.

H6 There is a positive correlation between job involvement and life satisfaction.

### 2.9 Job satisfaction and life satisfaction

The effects of job satisfaction on life satisfaction are supported in the extant literature (Steel [[102], [103], [104]], based on the spillover model. The spillover theory suggests a positive relationship between job satisfaction and life satisfaction based on the principles of “generalization of beliefs and attitudes, conditioning, and cognitive dissonance” [105]. Consistent with the spillover theory, Unanue et al. [106] examined 636 Chilean working adults and reported that higher job satisfaction enhanced life satisfaction. Cerci and Dumludag [107] also surveyed 1, 215 respondents recruited from nine public universities in Turkey and observed a positive and significant relationship between job satisfaction and life satisfaction. Using the spillover theory, Diener and Tay [108] opined that job satisfaction is a significant indicator of workers’ well-being and also influences life satisfaction because “the job is an important part of adult daily life, people who enjoy their jobs will report greater overall satisfaction with their lives” [109]. From the above evidence, the following hypothesis was developed:

H7 There is a positive correlation between job satisfaction and life satisfaction.

### 2.10 Organisational commitment, job involvement, job satisfaction and life satisfaction

According to Gunlu et al. [110], a committed employee is prone to exhibit positive work attitudes and behaviours. Several studies have found a positive relationship between organizational commitment and several work outcomes [[111], [112], [113]]**.** For example, in the studies of Lambert et al. [112] and Caillier [114], organizational commitment correlated positively with job involvement. In other studies, organizational commitment was positively related to job satisfaction [111,112,115]. De Cuyper et al. [113] have also reported a positive and significant relationship between organizational commitment and life satisfaction in the study of 568 employees from divisions of eight Belgian companies. Based on the SET, the study contends that when employees have a strong commitment to their organization arising from the impression of fair treatment or feel valued and respected, they are likely to reciprocate by spending more effort, time, and energy on their job, feel satisfied with the job and life [116,117]**,** experience work satisfaction, seeing it as a gratifying component of their reciprocal relationship [118]**,** and satisfaction with life because committed employees might experience a sense of fulfilment, support, and positive exchanges that spill over into their personal lives [119]**.** Consequently, the following hypotheses were developed:

H8 There is a positive correlation between organizational commitment and job involvement.

H9 There is a positive correlation between organizational commitment and job satisfaction.

H10 There is a positive correlation between organizational commitment and life satisfaction.

**Fig. 1:**
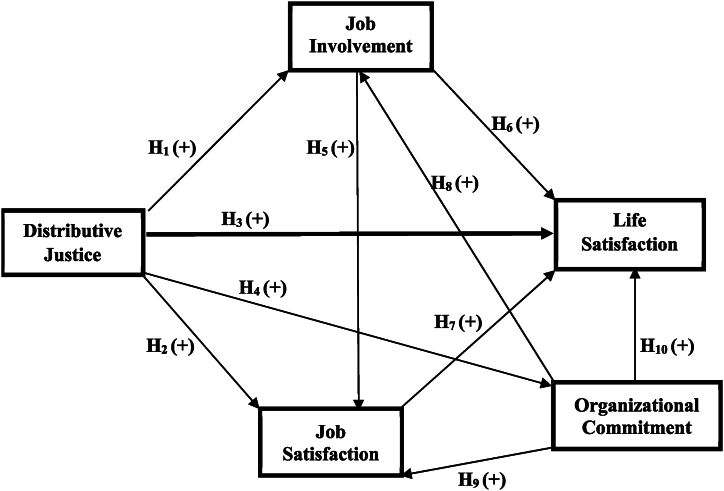
Conceptual framework.

The model below illustrates the expected relationship between the variables (Fig. 1).

## 3 Methodology

### 3.1 Sample and procedure

The population for the study consisted of approximately 5123 employees working in 793 licensed hotels ranging from five-star to budget facilities located in the Greater Accra Region of Ghana [120]. The sample for the study was selected using a multi-stage sampling technique. Firstly, hotels were selected using stratified sampling, and secondly, individual cases were sampled from the strata using the quota sampling technique to make up the sample for the study. Based on a confidence level of 95% and a confidence interval of five, the sample size for the study was estimated to be 358. Of 50 invitations sent to human resource managers, 28 star-rated facilities agreed to participate in the study, and packets of paper-and-pencil questionnaires that took on average 10 min to complete were hand-delivered to the human resource departments of the hotels in February 2019 to oversee the distribution to all employees, ensuring fair representation of all units and departments in the facilities [121]. Heads of human resource departments were instructed to ensure voluntary participation and assure employees of anonymity and confidentiality. Out of 358 questionnaires administered by student research assistants, 340 were retrieved from the facilities in April 2019, of which 19 were discarded due to incompleteness, leaving 321 used in the data analysis, giving a response rate of 89%. The study was approved by the university's Research and Ethics Review Committee, and it fulfilled the provisions and principles of the Declaration of Helsinki for research on human subjects.

### 3.2 Measure*s*

**Job Satisfaction** was measured using 5-items adopted from Warr et al. [122]. Sample items include “The work I do on my job is meaningful to me”, “All in all, I am satisfied with my job”. **Job Involvement** as measured using 8-items adopted from Kanungo [41]. Sample items include “I live, eat and breathe my job”, “the most important things that happen to me involve my present job”. **Distributive justice** was measured using Niehoff and Moorman [123] 4-item subscale of organizational justice. An example of the items includes ‘‘my work schedule is fair’‘. **Life satisfaction** was measured using the scale of Diener et al. [108], whereas **organizational commitment** was measured using an adopted scale developed by Porter et al. [124]. An example of the items included “I really care about the future of this hotel”. The participants rated all items of the constructs on a 5-point Likert scale ranging from 1 (*strongly disagree*) to 5 (*strongly agree*).

### 3.3 Data analysis procedure

First, the demographic profile of the research participants was summarized using descriptive statistics. Second, partial least squares-based structural equation modelling (PLS-SEM) was used to assess the research framework and its related hypotheses [125]. In line with a recommendation by Hair et al. [125], the measurement model was first assessed before evaluating the structural model. The PLS algorithm was used to assess the features of the measurement model, including model fit, discriminant validity, adjusted R^2^ and inner VIF values. Besides, the study's structural model was analysed by applying bootstrapping sampling (10,000 re-samples) to determine the direct and indirect path coefficients and their respective significance levels. Bagozzi et al.‘s [126] method was used to test Common-Method Variance (CMV). As shown in Table 2, the highest correlation between any two constructs is 0.543 < 0.90 [125] (correlation between OC and LS). Hence, the data did not have an issue with CMV.

**Table 1:** Demographic profile of sample.

Characteristic		Frequency	Percent
Gender	Male	177	53.6
	Female	144	43.6
Age	≤30 yrs.	168	52.3
	31–50 yrs.	147	45.7
	≥51 yrs.	6	1.9
Marital Status	Married	185	57.6
	Single	136	42.4
Level of Education	Secondary Education	140	43.6
	Undergraduate Education	162	50.5
	Post Graduate Education	19	5.9
Years of Experience	≤5 yrs.	199	62.0
	6–10 yrs.	67	21.0
	≥15 yrs.	55	17.0
Department	Housekeeping	62	19.4
	Food and Beverage	61	19.1
	Front Office	48	14.8
	Kitchen	46	14.2
	Administration	61	19.0

**Table 2:** Validity and reliability and cross loadings of constructs.

Constructs and Items	Loadings	AVE	CR	CA
Distributive Justice		0.523	0.814	0.700
*DJ1*	0.701			
*DJ2*	0.708			
*DJ3*	0.710			
*DJ4*	0.812			
Job Involvement		0.509	0.861	0.806
*JI3*	0.725			
*JI4*	0.705			
*JI5*	0.707			
*JI6*	0.741			
*JI7*	0.737			
*JI8*	0.760			
Job Satisfaction		0.590	0.850	0.767
*JS1*	0.787			
*JS2*	0.827			
*JS3*	0.872			
*JS4*	0.757			
Life Satisfaction		0.621	0.891	0.845
*LS1*	0.760			
*LS2*	0.866			
*LS3*	0.853			
*LS4*	0.794			
*LS5*	0.743			
Organisational Commitment		0.565	0.866	0.806
*OC1*	0.701			
*OC3*	0.769			
*OC4*	0.794			
*OC5*	0.803			
*OC6*	0.702			

## 4 Results

### 4.1 Demographic profile of respondents

The sample was slightly dominated by male employees (53.6%), with females constituting 43.6%. Exactly 57.6% of the respondents were unmarried and largely (50.5%) holders of undergraduate degrees. A little over half (52.3%) of the respondents were aged 30 years or less, with 62% reporting work experience ranging between 1 and 5 years working in departments such as housekeeping (19.4%), food and beverage (19.1%), front office (14.8%), kitchen (14.2%), and administration (19.0%) (Table 1).

### 4.2 Assessment of measurement model

The results relating to the outer model evaluation are set out in Table 2, Table 3. To establish convergent validity and reliability for the constructs used in the study, items with loading values of less than 0.7 were removed. For instance, two items of job satisfaction and job involvement, 4 items of distributive justice, and 1 item of organizational commitment were deleted. As shown in Table 2, Cronbach's Alpha for distributive justice, job involvement, job satisfaction, life satisfaction and organizational commitment exceeded the suggested threshold of 0.70 [127], thereby confirming the reliability of all the measures. Similarly, both the composite reliability and average variance extracted values exceeded the permissible thresholds of 0.70 and 0.50 respectively, thus affirming the reliability and validity of the model's latent variables [128,129].

**Table 3:** Q^2^ and discriminant validity (fornell-larcker criterion).

	Q^2^	DJ	JI	JS	LS	OC
**Distributive Justice (**DJ**)**		0.723				
**Job Involvement (**JI**)**	0.006	0.121	0.713			
**Job Satisfaction (**JS**)**	0.115	0.192	0.408	0.768		
**Life Satisfaction (**LS**)**	0.207	0.406	0.329	0.306	0.788	
**Organisational Commitment (**OC**)**	0.175	0.379	0.387	0.452	0.541	0.751

Three procedures were used to test the model's discriminant validity. First, Fornell and Larcker's [130] technique was examined (Table 3). As shown in the Table, the square root of the AVEs of each construct in the matrix diagonal is higher than the related correlation in the corresponding rows and columns [125], thereby demonstrating the quality of the reflective model. Secondly, the Heterotrait-Monotrait proportion of relationships (HTMT) criteria was estimated for each pair of reflective constructs based on the item correlations [131]. As shown in Table 4, the results from the correlations between pairs of constructs are less than the threshold values of HTMT = 0.90 [131]. Therefore, the discriminant validity of the constructs is confirmed. Finally, the cross-loading values of reflective construct indicators were evaluated. As shown in Table 2, all the indicators of the reflective measurement model met the cross-loading assessment criteria, thereby providing evidence for the discriminant validity of the reflective measurement model [125].

**Table 4:** Heterotrait-monotrait ratio (HTMT).

	DJ	JI	JS	LS	OC
**Distributive Justice (**DJ**)**					
**Job Involvement (**JI**)**	0.197				
**Job Satisfaction (**JS**)**	0.237	0.513			
**Life Satisfaction (**LS**)**	0.497	0.401	0.376		
**Organisational Commitment (**OC**)**	0.477	0.474	0.567	0.647	

### 4.3 Assessment of structural model

The standardized root means square residual (SRMR) value was used to determine the overall model fit [132]. For the study, the SRMR value was 0.071 < 0.08, indicating a good model fit [133] (Table 5). The R^2^ criterion was used to assess the predictive power of the structural model [134] (Chin, 1998). The assessment of the endogenous constructs' predictive power reveals small to very strong R^2^ values. The results show that distributive justice accounted for a 1.3% variance in job involvement and an 18% variation in job satisfaction. Similarly, 36.5% of the variance in life satisfaction is explained by a combination of distributive justice, job satisfaction, organizational commitment and job involvement. Finally, distributive justice, job satisfaction and job involvement accounted for 32.8% of the variance in organizational commitment (Table 5). The Stone-Geisser's Q2 Test [135,136] was used to evaluate the predictive validity of the exogenous latent variables in the model using blindfolding. As shown in Table 3, all Q^2^ values were significantly above zero, confirming that the exogenous constructs have high predictive relevance [134]. Cohen's [137] *f*^*2*^ was used to assess the effect size of the main exogenous construct. An analysis of the results suggests that the magnitude of the effect of distributive justice on job involvement (*f*^*2*^ = 0.116) and job satisfaction (*f*^*2*^ = 0.132), life satisfaction (*f*^*2*^ = 0.173) and organizational commitment (*f*^*2*^ = 0.118) met the effect threshold of medium size. Likewise, the effect of job involvement on job satisfaction (*f*^*2*^ = 0.173) and life satisfaction (*f*^*2*^ = 0.121) met a medium effect. The effect of organizational commitment on job involvement (*f*^*2*^ = 0.167), job satisfaction (*f*^*2*^ = 0.110) and life satisfaction (*f*^*2*^ = 0.148) met a medium effect. Finally, of job satisfaction on job satisfaction (*f*^*2*^ = 0.401) met a large effect size.

**Table 5:** Summary of fit and R^2^ of structural model.

Construct Coefficient of Determination (R^2^)	R^2^	Adjusted R^2^
**Job involvement**	0.016	0.013
**Job satisfaction**	0.185	0.180
**Life satisfaction**	0.363	0.355
**Organizational commitment**		
Model Fit	**Value**	
**SRMR**	0.071	

In order to control the effects of extraneous variables, the demographic variables of the respondents i.e., age, gender, education, living status, marital status and work experience were included in the model as control variables. Except for work experience, all the other five variables did not influence the dependent variable, life satisfaction. As shown in Table 6, the life satisfaction of hotel employees increases with longer periods of working experience in the hotels.

**Table 6:** Path coefficient and hypothesis assessment.

Paths	β	t-Values	p-Values	Results
Distributive Justice - > Job Involvement	0.133	1.963	**0.050**	Supported
Distributive Justice - > Job Satisfaction	0.164	3.337	**0.001**	Supported
Distributive Justice - > Life Satisfaction	0.238	5.212	**0.000**	Supported
Distributive Justice - > Organisational Commitment	0.379	7.167	**0.000**	Supported
Job Involvement - > Job Satisfaction	0.275	5.008	**0.000**	Supported
Job Involvement - > Life Satisfaction	0.140	2.381	**0.017**	Supported
Job Satisfaction - > Life Satisfaction	0.030	0.452	0.651	Not Supported
Organisational Commitment - > Job Involvement	0.398	6.630	**0.000**	Supported
Organisational Commitment - > Job Satisfaction	0.333	5.218	**0.000**	Supported
Organisational Commitment - > Life Satisfaction	0.384	6.885	**0.000**	Supported
**Control Variable**	**Life Satisfaction**			
Age	0.289			
Gender	0.41			
Education	0.998			
Living Status	0.717			
Marital Status	0.826			
Work Experience	0.003			

As shown in Table 6, **H**_**1**_, **H**_**2**_**, H**_**3,**_ and **H**_**4**_ were supported by the data as distributive justice has a significantly positive effect on job involvement (β = 0.133; t = 1.963; p = .050), job satisfaction (β = 0.164; t = 3.337; p = .001), life satisfaction (β = 0.236; t = 5.093; p = .000) and organizational commitment (β = 0.379; t = 7.167; p = .000). **H**_**5**_ and **H**_**6**_ were also supported as job involvement has a positive and significant effect on job satisfaction (β = 0.275; t = 5.008; p = .000) and life (β = 0.140; t = 2.381; p = .017). However, **H**_**7**_ was not supported since the effect of life satisfaction on job satisfaction was not significant (β = 0.030; t = 0.452; p = .651). Finally, **H**_**8**_**, H**_**9**_ and **H**_**10**_ were supported by the data as organizational commitment has a positively significant effect on job involvement (β = 0.398; t = 6.630; p = .000), job satisfaction (β = 0.333; t = 5.218; p = .000) and life satisfaction (β = 0.384; t = 6.885; p = .000).

## 5 Discussion

The study examines the interplay between and among distributive justice, job involvement, job satisfaction, organizational commitment and life satisfaction among 321 hotel employees selected from 28 star-rated accommodation facilities in the Greater Accra region of Ghana. In this study, structural PLS modelling was used to test 10 hypotheses. The results from the analysis support nine out of the ten hypotheses.

The results show distributive justice influences hotel employees job involvement and job satisfaction. These findings align with the SET theory [73] and are consistent with previous works on distributive justice and job involvement [100,84], job satisfaction [19]**,** and organizational commitment [92,90]. These findings suggest that hotel employees’ perception of fairness in the distribution of organizational outcomes encourages employees to become absorbed and satisfied with their jobs and committed to their organization. The SET theory suggests that distributive justice plays a pivotal role in shaping hotel employees' attitudes and behaviours since they perceive that they are receiving fair and just rewards for their efforts. Their perception of fairness fosters trust in the organization and its management and contributes to a more committed, satisfied, and productive workforce. The observed positive effect of organizational justice on life satisfaction is consistent with earlier studies [89,88], a confirmation of the propositions espoused in the spillover theory [74], suggesting that the happenings at the workplace affect the personal lives of employees. From the perspective of the spillover model, the findings suggest that when there is fairness at the workplace, employees are likely to experience reduced job-related stress and anxiety, higher levels of job satisfaction, a better work-life balance, positive emotions, and overall well-being [[138], [139], [140]] These positive experiences can spillover into their personal lives, resulting in fulfilling life satisfaction.

In support of spillover theory [74] and earlier studies, job involvement was found to influence hotel employees job satisfaction [96,98,99] and life satisfaction [[141], [142], [143]]. However, job involvement was a stronger predictor of job satisfaction compared to life satisfaction. The implication of this result is that involved hotel workers are most likely to experience higher levels of job satisfaction as well as life satisfaction. On the premise of spillover theory, the findings suggest hotel employees job involvement enhances their sense of engagement and commitment to work. This heightened engagement can result in positive emotions and a sense of accomplishment, contribute to the development of psychological capital, including self-efficacy, optimism, hope, and resilience [144], and improve performance, leading to positive feedback from supervisors and colleagues. These positive experiences, emotions, and attitudes related to high job involvement can spill over into both personal lives and job satisfaction, contributing to overall well-being and satisfaction.

In contrast with earlier studies [106,107] and expectations, the results of the present study did not support a relationship between job satisfaction and life satisfaction. The possible explanation for the insignificant relationship between job satisfaction and life satisfaction could be the lack of positive spillover from employees’ job experiences to their lives in the case of job satisfaction. For instance, employees in the hotel sector in Ghana encounter multiple stresses in their everyday lives, including financial challenges, health issues, and an inadequate standard of living. These pressures may have permeated their work environment and hindered the positive spillover from job satisfaction to life satisfaction. The findings suggest that the use of spillover theory to elucidate the relationship between job and life satisfaction is not universal but varies significantly depending on the individual involved, the condition, and the context. Furthermore, in Ghana, hotel employees' life satisfaction is influenced by multiple factors, and job satisfaction alone may not be sufficient to significantly enhance overall life satisfaction. In line with previous studies, organizational commitment was found to positively influence hotel employees job involvement [112,114], suggesting that hotel employees who are committed to their organizations are most likely to be highly involved in their jobs; job satisfaction [92,115], Indicating that as commitment increases, so will the job satisfaction levels of hotel employees and life satisfaction [113], suggesting that employees committed to their hotels are likely to report higher levels of life satisfaction. However, the influence of organizational commitment was stronger for life satisfaction relative to job involvement and job satisfaction. Using the SET, the findings indicate that hotel employees evaluate their commitment towards the organization as a gain and are motivated to maintain or enhance their commitment to the organization because they perceive these gains as valuable in relation to their job involvement, job satisfaction, and life satisfaction.

## 6 Theoretical implications

In view of the paucity of literature on the influence of distributive justice on the work attitudes of hotel workers, this study provides additional insight on the applicability of the spillover model to explain the interrelationships between the workplace and the personal circumstances of employees. According to Lambert et al. [27], job involvement is an important construct that requires further probe. In this regard, the current study extends the literature on the factors that influence the job involvement of employees within the hotel sector from a developing country perspective. Theoretically, the result of the study offers support for the link between distributive justice and job satisfaction, job involvement, life satisfaction, and organizational commitment. Given that the study was conducted in a context where the practice of distributive justice is less appreciated, the significant findings suggest there might be other influential factors behind the positive responses of research participants worth examining. The study relied on social exchange and spillover theories to explain the hypothesized relationships. Using social exchange theory, the study expands our understanding of how distributive justice affects employees' job satisfaction, organizational commitment, and job involvement. Similarly, the spillover theory was used in the study to explain the relationship between distributive justice and life satisfaction, as well as the relationship between job satisfaction and life satisfaction. The findings suggest that the positive perception of managers' fairness by employees could manifest in positive spillover behaviours, including life and job satisfaction.

## 7 Practical implications

The results of the study have useful practical implications for hotel operators and policymakers. The significant association between job satisfaction and life satisfaction means that the spillover hypothesis is significant in the case of this study. Thus, for the hotel employees, their job experiences spill over into other spheres of life. Consequently, hotel operators and managers must take steps to design work that would enhance employees' job satisfaction. For instance, managers are encouraged to empower employees to make decisions within their areas of responsibility [145]. This not only reduces the need for constant supervision but also gives employees a sense of autonomy and ownership in their roles. Additionally, managers must establish open channels of communication with staff, which must be used to regularly seek feedback, and the information so obtained can be used to improve work processes and job conditions. The result of the study also provides satisfactory evidence in support of the effect of distributive justice on the life satisfaction and organizational commitment of employees. The implication of this finding calls for reforms in the practices of distributive justice in hotels to provide assurance of fairness among employees. Managers, for instance, must develop and communicate standard procedures for how resources and outcomes will be distributed in the organization. Secondly, they must stick to the full implementation of standard procedures to avoid the tag of discrimination. Managers can further promote distributive justice by promoting transparent compensation policies [146,147]. Management must take steps to educate employees on how their salaries and bonuses are determined, and further communicate these policies openly and regularly, and ensure that they are enforced uniformly across the organization. They must also take steps to implement a wage system that is equitable for all workers and reflects their abilities, tasks, and performance [148]. Additionally, managers are encouraged to create and implement a fair decision-making process that reduces bias and serves the needs of all employees. Finally, managers are encouraged to offer employees equal opportunity for skill development to advance in their careers [149]. This not only promotes fairness but also provides a path for employees to enhance their value and earn higher compensation over time. To enhance employee job involvement and organizational commitment, managers should ensure that employees have a comprehensive comprehension of their roles, responsibilities, and the overarching objectives of the organization. This may be achieved via several means, such as conducting frequent feedback sessions, organizing one-on-one meetings, and facilitating team meetings [150]. These platforms serve as opportunities to address any inquiries, apprehensions, or uncertainties that may arise, while also offering direction and support to individuals within the team. Managers can also ensure effective employee job involvement and commitment through the fostering of a positive and inclusive work environment through teamwork, a healthy work-life balance, and social interaction [151].

## 8 Limitations and future research directions

Like other research works, this study has limitations that must be acknowledged. First, the study was restricted to a sample of hotel workers selected from Accra, raising the possibility of sample bias. Second, the sample size used in the data analysis was less than the estimated number of respondents required for the study. Third, the data used for analysis was gathered by means of a cross-sectional design, which increases the possibility of common method variance. Fourth, the research sample was selected using the quota sampling technique. All these concerns limit the representativeness and generalizability of the results of the study to other hotel employees in Ghana and elsewhere. It is recommended that future studies include a larger sample size, cover a broader population, and use probability sampling techniques.

Future research has the potential to enhance the existing model by including a mediation-moderation framework. This may be achieved by systematically finding and adding mediators and moderators via an extensive review of relevant literature. For instance, in the case of moderators, job tenure might be used to moderate the nexus between job involvement and job satisfaction since employees with longer tenure might experience different levels of job satisfaction compared to those who are newer to the job. Personality traits such as neuroticism and extraversion could be used to moderate the association between job involvement, job satisfaction, and life satisfaction, given that some personality traits may amplify or dampen the effects of the proposed association. In terms of mediators, future studies can explore psychological empowerment as a mediator between distributive justice and life satisfaction, given that when employees feel empowered in their work, they may experience greater life satisfaction. Additionally, work-life balance could be used to mediate the nexus between job involvement and life satisfaction, on the premise that when employees can balance their work and personal lives effectively, it may positively impact their overall life satisfaction. Finally, job autonomy can be explored as a mediator between job involvement and job satisfaction, given that higher job autonomy may lead to increased job involvement, which in turn may enhance job satisfaction.

## Funding statement

This study did not receive any specific grant from funding agencies in the public, commercial, or not-for-profit sectors.

## Data availability statement

Data would be made available on request.

## Additional information

No additional information is available for this paper.

## CRediT authorship contribution statement

**Christopher Mensah:** Writing – review & editing, Writing – original draft, Supervision, Project administration, Formal analysis, Conceptualization. **Edem M. Azila-Gbettor:** Writing – review & editing, Writing – original draft, Methodology, Formal analysis, Conceptualization. **Melody E. Appietu:** Writing – original draft, Project administration, Data curation, Conceptualization.

## Declaration of competing interest

The authors declare that they have no known competing financial interests or personal relationships that could have appeared to influence the work reported in this paper.
